# Remedies and Their Uses

**Published:** 1907-01-19

**Authors:** 


### Remedies and their Uses.

### Exodin.

This is a new purgative manufactured by Schering. It is somewhat similar to "purgatin," being an oxyanthraquinone derivative (diacetyl-rufigallic acid tetramethyl ether). It is a yellow, tasteless, inodorous powder insoluble in water and sparingly soluble in alcohol. It is pleasant to take as it it has no tendency to upset the stomach; the action is free from any griping, and is as a rule single, producing a soft water evacuation. The effect of the drug sets in as a rule after six to eight hours. The value of exodin is chiefly in cases of habitual chronic constipation in otherwise healthy persons; it is especially useful in the constipation associated with the earlier months of pregnancy. Its efficacy appears to be scarcely impaired by repeated administration. Exodin is supplied in 0.5 gramme (7½ grain) tablets and the dose is from one to three tablets; they may be broken up if preferred and the powder suspended in water. For children one tablet is enough.

### Hypnal, or antipyrin-chloral hydrate, as prepared also by Meister Lucius and Bruning, is an essentially safe and certain hypnotic in many forms of mental disease; as a sedative in acute mania or early delirium tremens it has produced quiet sleep, and will be found of the greatest service in the treatment of restless cases of chorea. It is soluble in water, and, therefore, is very simple to dispense, or it may be ordered in cachets or powders, gr. 20 for single administration or 10 if repeated. Hypnal is free from depressant after-effects and does not tend to produce symptoms of collapse.

